# Small Molecule Inhibitors of HSF1-Activated Pathways as Potential Next-Generation Anticancer Therapeutics

**DOI:** 10.3390/molecules23112757

**Published:** 2018-10-24

**Authors:** Chiranjeev Sharma, Young Ho Seo

**Affiliations:** College of Pharmacy, Keimyung University, Daegu 42601, Korea; 1chiranjeevsharma1@gmail.com

**Keywords:** HSF1, HSPs, HSF1 inhibitors, anticancer agents

## Abstract

Targeted therapy is an emerging paradigm in the development of next-generation anticancer drugs. Heat shock factor 1 (HSF1) has been identified as a promising drug target because it regulates several pathways responsible for cancer cell growth, metastasis, and survival. Studies have clearly demonstrated that HSF1 is an effective drug target. Herein, we provide a concise yet comprehensive and integrated overview of progress in developing small molecule inhibitors of HSF1 as next-generation anticancer chemotherapeutics while critically evaluating their potential and challenges. We believe that this review will provide a better understanding of important concepts helpful for outlining the strategy to develop new chemotherapeutic agents with promising anticancer activities by targeting HSF1.

## 1. Introduction

**Figure 1:**
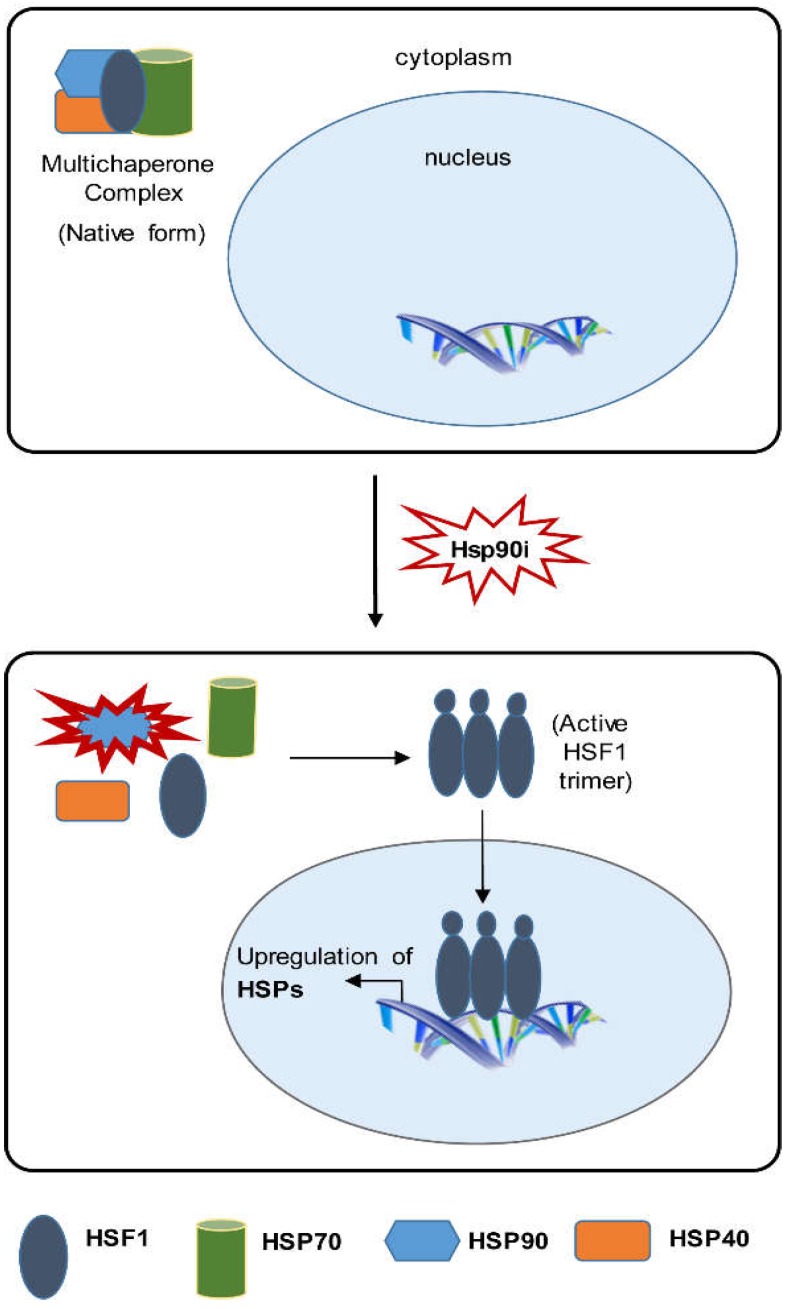
HSP90 inhibitors disrupt the multichaperone complex of HSF1 with other HSPs, subsequently leading to phosphorylation, nuclear translocation, and binding to heat shock elements in DNA, resulting in upregulation of HSPs for cancer cell survival.

Cancer is one of the leading causes of death worldwide. It is potentially fatal with severe health and economic impacts [1]. The total annual economic cost of cancer in 2010 was estimated to be approximately 1.16 trillion US dollars. According to WHO, 9.6 million people would die from cancer in 2018. Thus, there is an urgent need to find alternative strategies to reduce the cancer burden. An actively pursued therapeutic approach that has emerged as a promising strategy recently is to target heat shock proteins (HSPs). It is apparent that cancer cells will experience adverse effects and proteotoxicity due to several intrinsic and extrinsic factors. To withstand proteomic chaos and protect themselves from thermal stress, oxidative damage, and hypoxia, cells can adapt a compensatory stress response mechanism by increasing levels of HSPs. HSPs are molecular chaperones that ensure protein homeostasis by proper folding of proteins or refolding of misfolded proteins, thereby protecting cells from protein misfolding and aggregation. Cancer cells differ from normal cells as they have higher need for chaperone proteins for survival. Stress response is known to play a vital role in preserving protein homeostasis [2,3]. Several HSP inhibitors have shown promise for the treatment of cancer. In this regard, HSP90 inhibitors have been extensively studied. We have also reported several novel HSP90 inhibitors based on resorcinol and 1,3,5-triazines [4,5,6,7]. Several HSP90 inhibitors are currently tested in clinical trials [8]. Despite the ongoing preclinical and clinical studies with good mechanistic rationale, insights taken from the published data suggest that the use of HSP90 may be limited due to the fact that HSP90 inhibition activates heat shock factor 1 (HSF1) (Figure 1) [9,10]. HSF1 is a potentially better target for cancer therapy than HSP90 and several other HSPs [11].

HSF1 is a master regulator of transcriptional responses to proteotoxic stress. It is crucial in cancer as it enables cancerous cells to adapt to oncogenic stress and drastic conditions [12]. It also induces the expression of other cytoprotective stress proteins such as HSP40 and HSP70. HSF1 is also known to induce multidrug resistance genes against both cisplatin and carboplatin treatment [13].

Targeting only part of the heat shock response (HSR) or individual HSP inhibition might be ineffective as an anticancer strategy. A better rationale would be to inhibit HSF1, which lies in the core of HSR to develop inhibitors that are more potent. A number of small molecule activators and inhibitors of HSF1 have been found to be able to regulate HSR [14,15,16]. The initial compound reported as a small molecule HSF1 inhibitor had a natural origin. Thereafter, a plethora of high-throughput, phenotypic, and in silico screening studies have been performed, leading to the identification of many good candidates of synthetic and natural origin. Another interesting approach used to develop HSF1 inhibitors was synthetic modification of known structures to design effective drug agents. However, there is still an acute shortage of clinically suitable chemical agents that could progress beyond pre-clinical studies for therapeutic use.

In this review, we will first introduce the structure and role of HSF1 in cancer, with an emphasis on its transcriptional potential with compensatory role to sustain tumor cell survival and growth during heat shock. We will then discuss small molecule inhibitors of HSF1 and mechanisms involved in their inhibition of HSF1. Finally, we will conclude the review by addressing some issues that should be answered before successfully targeting HSF1 for the development of anticancer drugs.

## 2. Structure and Function of HSF1

HSF1 is an evolutionarily highly conserved transcription factor essential for cancer cell survival [17]. It regulates the complex defense mechanism of cancer cells to protects them against heat shock due to increased temperature or other stresses [18]. HSF1 belongs to the HSF family that can be grouped into five major family members, namely, HSF1, HSF2, HSF3, HSF4, and HSF5 [19]. HSFs are believed to play a significant role in cancer survival by either activating or repressing their target genes [20]. Furthermore, there are two more isoforms of human HSFs, HSFX and HSFY. They play a role in human spermatogenesis [19]. Among all HSFs, HSF1 is the main transcriptional regulator of HSR. Hence, we will discuss the structure and function of HSF1 in detail.

**Figure 2:**
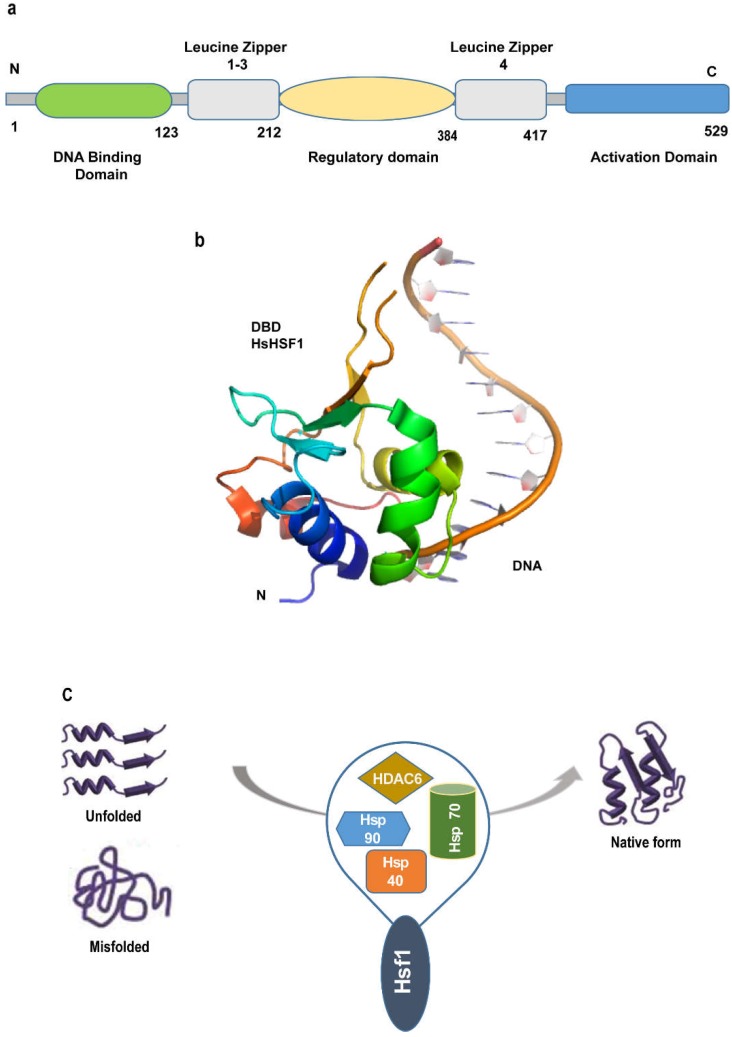
(**a**) Domain structure of HSF1; (**b**) Crystal structure of Human HSF1 with DNA (PDB: 5D5U); (**c**) HSF1 with the help of co-chaperones can prevent toxic buildup of unfolded proteins and assist in refolding of misfolded proteins to prevent aggregation and promote cellular protection.

HSF1 is a 57 kDa cytoplasmic protein whose overexpression is related to increased malignancy and mortality in many cancer types, including breast, prostate, lung, kidney, and pancreas cancers [21]. The structure of HSF1 consists of four major functional domains: a DNA binding domain (DBD), an oligomerization domain, a regulatory domain, and a transactivation domain. Although the crystal structure of full-length HSF1 is currently unavailable, the co-crystal structure of human HSF1 DBD with DNA has been reported [22] (Figure 2). The N-terminal of HSF1 consists of DBD which binds to the major groove of DNA by recognizing nGAAn sequences. The pentamers of these sequences repeated in head to tail fashion (for example, 5′-nGAAnnTTCnnGAAn-3′) are known as heat shock elements (HSE).

The oligomerization domain consists of leucine zippers that are responsible for HSF1 trimerization. Leucine zippers facilitate the oligomerization of HSF1 monomers by intermolecular disulfide bonding, resulting in three-stranded coiled-coil structures. The regulatory domain occurs between leucine zipper 1–3 and leucine zipper 4. The regulatory domain and leucine zipper represent the site of posttranslational modification such as phosphorylation, acetylation, and sumoylation that are responsible for both activating and repressing transcriptional functions as summarized in Table 1. Finally, C-terminal consists of an activation domain that is responsible for transcriptional activation of HSF1 [23].

HSF1 exists as an inactive form under unstressed conditions. It is complexed to HSP40, HSP70, and HSP90. It has been suggested that cytoplasmic deacetylase HDAC6 is also involved in a multichaperone complex with inactive HSF1 [10]. The molecular chaperones HSP90 and HSP70 play a significant role in the molecular mechanism and repression of transcriptionally active HSF1.

HSP90 terminates the HSR by removal of HSF1 trimers from HSEs in DNA [9]. In contrast to the binding profile of HSP90, endogenous HSP70 shows strong interaction with full-length as well as with most of the C-terminal truncated and deleted mutants of HSF1. Thus, HSP70 provides stress-sensitive repression of the HSR in mammalian cells via major interaction with regulatory domain.

Stress or heat shock can dissociate HSF1 from chaperones. The monomeric inactive HSF1 is activated by phosphorylation [24]. Phosphorylated HSF1 is then trimerized and translocated into the nucleus. It binds to the heat shock element in DNA and initiates HSR by upregulating HSP27, HSP40, HSP70, and HSP90. HSF1 also increases mRNA translation under stress which increases the overall protein level, thus enhancing the chaperone capacity and promoting tumorigenesis. It also promotes refolding and degradation of proteins that are crucial in malignant cells (Figure 2). It also plays a critical role in cell survival when it is under stress by mediating the stress response. Thus, it qualifies as a promising drug target for anticancer therapy.

## 3. Natural HSF1 Inhibitors

HSF1 inhibitors of natural origins are summarized in Table 2. The first HSF1 inhibitor quercetin (**1**) came from nature as a structurally simple natural product. Quercetin belongs to the flavone family. It is famous for its various biological activities, including its ability to inhibit heat induced HSP70 expression. In 1990, it was reported that it could inhibit HSP synthesis induced by sodium arsenite and l-azetidine 2-carboxylic acid as heat shock at the level of mRNA accumulation [25]. Quercetin inhibited the binding of HSF to the HSE, both in vivo and in vitro in COS-7 and COLO 320DM cells. However, the concentration required to inhibit HSF activation in vitro (100 μM) was five-fold higher than that required in vivo (500 μM), perhaps due to the higher concentrations of drugs required to access HSF1 in cytoplasmic extracts [26]. Pretreatment with quercetin could decrease the level of phosphorylated HSF1 in heat shocked cells compared to normal cells. Chemical crosslinking experiments and gel filtration data confirmed that it did not affect HSF1 trimerization. However, it could reduce phosphorylation of HSF1 [27]. Quercetin is also known to inhibit kinases. It can enhance HSP70 induction through phosphorylation of HSF1. Quercetin is also known to inhibit kinases. The Phase I clinical trial of quercetin confirmed that it could be safely administered by i.v. for antitumor activity by in vivo tyrosine kinase inhibition [28]. Recently, the effects of quercetin-loaded liposomes in rats with R3230 breast adenocarcinoma improved radiofrequency ablation-induced tumor destruction resulting in improved clinical outcomes. A protective role of quercetin in tumor ablation was highlighted with a mechanism involving HSP70 with HSF1 pathway in thermal ablation of solid tumors [29].

Akagawa et al. [30] isolated another natural product, Stresgenin B (**2**) from the culture broth of *Streptomyces* sp. AS-9. It has biological activities similar to quercetin. It can also inhibit heat-induced HSP gene expression. Stresgenin B showed moderate cytotoxic activities against several neoplastic cell lines at micromolar concentration It was found to be 4.9-times more potent than quercetin. It was also more water soluble than quercetin.

**Figure 3:**
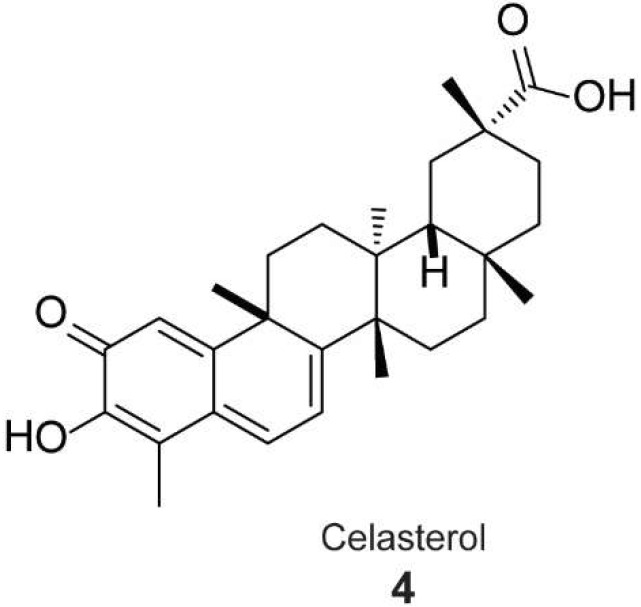
Chemical structure of celasterol.

Triptolide (**3**) is a biologically active diterpene triepoxide. It is another HSF1 inhibitor of natural origin. Westerheide et al. [31] identified triplotide as an inhibitor of HSF1during small molecule screening. It was originally isolated in 1972 from the Chinese plant *Triptergium wilfordii*. It can inhibit cell growth, induce apoptosis, and suppress transcriptional activation of NF-κB and activator protein-1 (AP-1) [31]. Interestingly, the same plant contains celastrols (**4**) which acts as inducers of HSR and cytoprotection (Figure 3) [32]. Triptolide is one of the most potent HSF1 inhibitors. Incubation of pancreatic cell lines, PANC-1 and MiaPaCa-2 cells with triptolide (50–200 nmol/L) significantly reduced cell viability. In vivo administration of triptolide (0.2 mg/kg/d for 60 days) also decreased pancreatic cancer growth and local-regional tumor spread [33]. Its mechanism of action is different from that of quercetin. It inhibits HSR by binding to the promoter of the HSP70 gene prior to induction at mRNA levels, suggesting that it does not affect phosphorylation, trimerization, or DNA binding of HSF1. Furthermore, in combination with triptolide, proteasome inhibitor bortezomib and HSP90 inhibitor NVP-AUY922 can enhance apoptotic effect. However, its clinical application is limited by its solubility and toxicity issues [34].

A cell-based screening with a library of diverse marketed and experimental drugs resulted in the identification of cantharidin (**5**) as a potent HSF1 inhibitor [35]. It inhibited heat shock-induced luciferase activity with an IC_50_ of 4.2 μM. Cantharidin chemically belongs to terpenoids. It can inhibit HSF1 transcriptional activity. In another cell-based screening of 760 structurally diverse natural compounds, Fisetin (**6**), a dietary flavonoid, was identified as an inhibitor of HSF1 [36]. It inhibits heat shock-induced luciferase activity in HCT-116 cancer cells with an IC_50_ of 14 μM. It further shows the antitumor effect of HSF1 inhibition in vivo in HCT-116 with a GI_50_ of 23 μM. The mechanism of inhibition by Fisetin was by inhibiting the binding of HSF1 to promoter region of HSP70.

Continued efforts to find better HSF1 inhibitors have prompted Santagata et al. [37] to integrate chemical and genetic approaches to screen more than 600,000 gene expression and more than 300,000 compounds. Rocaglamide A (**7**) belonging to a class of natural products called flavaglines was identified as a translation initiation inhibitor. It modulates tumor energy metabolism and selectively targets pre-malignant cells with early-stage oncogenic lesions. The mechanism for its effects on translation inhibition of HSF1 activity is believed to be subtle changes in translation activity involving eIF4A and/or other initiation factors.

Yoon et al. [38] also found a new inhibitor 2,4-Bis(4-hydroxybenzyl)phenol (**8**) by screening natural products isolated from several herbal medicines. This 2,4-Bis(4-hydroxybenzyl)phenol was isolated from rhizomes of *Gastrodia elata*. The mechanism of action of this new inhibitor was by inducing dephosphorylation of HSF1 at S326. Subsequently, the HSF1 protein stability is decreased, resulting in degradation and reduced levels of HSP27 and HSP70. It has a synergistic anticancer effect in combination with conventional drugs such as paclitaxel and cisplatin.

Recently, optimization of CL-43 (**9**), a cyclopentaneperhydrophenantrene derivative of cardenolide, was reported to be able to inhibit HSF1 activation in human colon cancer cells [39]. CL-43 can reduce the growth of cancer cells and their tumorigenicity. CL-43 showed the IC_50_ value of 479.2 ± 5.4 μM for HCT-116 cells. It also caused the death of 7.6 ± 0.5% of the cell at a concentration of 1 μM. The use of CL-43 in knockdown of HSF1 caused reduction of HSPs and resulted in weaker association of HSP90 with its co-chaperone CDC37, leading to partial depletion of other HSP90 client kinases, BTK, c-RAF, and CDK4. The authors speculated that CL-43 could interfere with the function of HSF1, similar to triplotide. However, its mechanism of action is currently unclear. Nevertheless, it showed enhanced potency with etoposide, cisplatin, and doxorubicin, suggesting that it could be used for various antitumor therapies.

## 4. Prodrugs of Natural HSF1 Inhibitors

**Figure 4:**
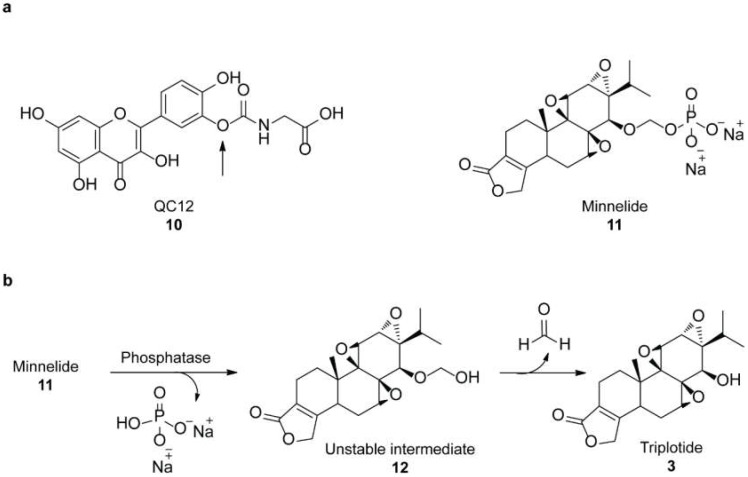
(**a**) Chemical structures of QC12 (the structure is hydrolyzed in-vivo at the arrowed oxygen to release quercetin and glycine) and Minnelide; (**b**) Phosphatases cleave the phosphate group from minnelide to yield an unstable O-hydroxymethyl intermediate (**12**) which degrades into formaldehyde and anticancer compound triptolide.

Solubility and toxicity issues limit the therapeutic use of some natural inhibitors of HSF1 such as quercetin and triptolide. A prodrug strategy has been used to improve their water solubility. Anticipating the release of a glycine and quercetin molecules, a water-soluble prodrug named QC12 (**10**) has been synthesized [40]. Expecting the phosphatase to cleave the phosphate group, a prodrug of triptolide named minnelide (**11**) has also been developed (Figure 4) [41]. Unfortunately, after phase I study of QC12, the oral bioavailability was found to be unsatisfactory. However, minnelide is as effective as the parent molecule triplotide in inhibiting tumor cells both in vitro and in vivo. Furthermore, it is non-toxic in mice even at higher doses. Minnelide is expected to begin Phase II clinical trial for treating gastrointestinal malignancies [42].

## 5. Synthetic HSF1 Inhibitors

A number of structurally diverse synthetic compounds have also been developed as HSF1 inhibitors. They are summarized in Table 3. Yokota et al. [43] reported a synthetic benzylidene lactam KNK437 (**13**) as an inhibitor of HSP induction in human colon carcinoma cells. It is more effective but less toxic than existing quercetin. It inhibits the activation of HSF1 and the interaction of HSF1 with HSE. However, it does not inhibit HSF1 phosphorylation. Later, KNK437 was also shown to be able to induce apoptosis and inhibit heat induced accumulation of HSPs, especially HSP27 and HSP70 [44]. KNK437 is not only effective against heat shock but also inhibits gene expression induced by chemical stress in amphibian cells. Furthermore, some reports strongly suggest that KNK-437 and bortezomib have synergistic effects on apoptosis. Thus, they can be used to treat multiple myeloma patients with adverse prognosis [45].

**Figure 5:**
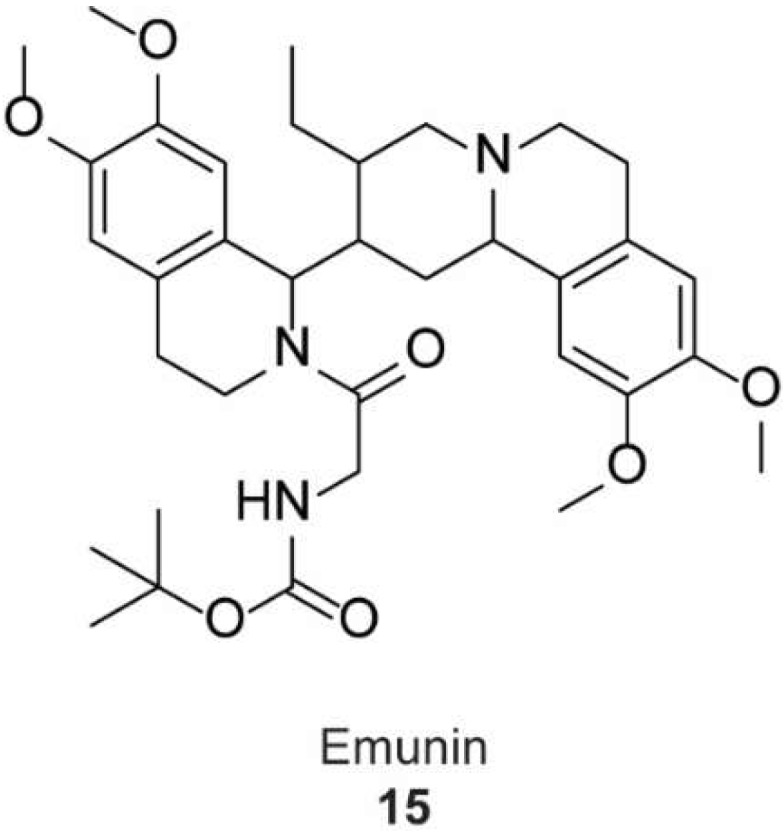
Chemical structure of emunin.

In 2006, Zaarur et al. [46] developed a high-throughput screen for small molecules that could inhibit induction of HSPs. They screened 20,000 compounds from different chemical libraries and identified NZ28 (**14**). They examined several analogues of NZ28 and found that the 2H-benzo(a)quinolizine tricyclic ring was a critical element for the activity. They found that emunin (**15**) (Figure 5) was practically nontoxic. These compounds were found to sensitize myeloma and prostate carcinoma cells to HSP90 and proteasome inhibitors. Their mechanisms of action are currently unclear.

In 2011, Yoon et al. [47] also screened a synthetic chemical library using luciferase reporter and found that KRIBB11 (**16**) had binding affinity to HSF1. This was the first molecule that could directly target HSF1. To confirm this, biochemical purification approach was employed using biotinylated KRIBB11. HSF1 protein associated with biotinyl-KRIBB11 was revealed by western blot analysis, confirming that HSF1 was the binding target of KRIBB11. The binding of KRIBB11 to HSF1 can disrupt the recruitment of p-TEFb protein and activate the transcription of heat shock proteins. It is commercially available now. It can also be successfully combined with HSP90 inhibitors.

Results from reporter-based high-throughput screening of over 100,000 compounds helped identify another HSF1 inhibitor named PW3405 (**17**) with diverse chemical functionality [48]. PW3405 showed nanomolar potency and relatively low cytotoxicity toward multiple cancer cell lines. It inhibited phosphorylation of HSF1, followed by reduced expression of HSP proteins. In a recent in silico screening of a large library of compounds, thiazole fused acrylamides were identified as potent HSF1 inhibitors [49]. Furthermore, discriminating cell-based screening assay and systematic analysis resulted in the development of I_HSF_115 (**18**) as a new inhibitor targeting human transcription factor. I_HSF_115 is the second molecule after KRIBB11 that can bind directly to HSF1.

Recently, Rye et al. [50] discovered 4,6-disubstituted pyrimidines using high-throughput phenotypic screening. Despite the kinase selectivity profile of the identified bisamide (**19**) and its ability to affect HSF1 pathway, it is unsuitable for clinical use due to its high clearance in mouse pharmacokinetic experiments. Cheeseman et al. [51] also discovered a new HSF1 inhibitor bisamide (CCT251236) (**20**) using an unbiased phenotypic screening. It was found to be orally bioavailable and efficacious in human ovarian cancer. Post-translational phosphorylation and kinase inhibition due to CCT251236 are believed to cause HSF1-mediated HSP72 induction inhibition. This study led to the identification of pirin as a high affinity molecular target. The role of pirin in modulating the HSF1 pathway is currently under investigation. Moreover, this study led to the identification of another HSF1 inhibitor CCT361814 with bioavailability features compatible for clinical use [52]. The inhibition of HSR by CCT361814 in multiple myeloma cells is expected to enter early phase clinical trials. In continuation of the identification of pyrimidine and bisamide as HSF1 inhibitors, a library of α-acyl aminocarboxamides was created to develop new inhibitors [53]. The authors aimed to investigate the structure-activity relationship between α,β-unsaturated carbonyl moiety and HSF1 related activity. A four-component Ugi reaction using an aldehyde, an amine, an isocyanide, and a carboxylic acid resulted in a library of α-acyl aminocarboxamides. Desired structures were synthesized and investigated. The most potent compound, aminocarboxamide (**21**) shown in Table 3, resulted in the loss of total HSF1 protein expression due to destabilization and impaired translation of HSF1 mRNA.

Despite significant progress in the development of novel HSF1 inhibitors, the specificity of mechanism of action has limited the therapeutic use of these drugs. Recent reports on aminocarboxamide and bisamide inhibitors have revealed the role of PIKK family, CDK9, and Pirin as key molecular targets to inhibit the HSF1 stress pathway [50,52,53]. New studies should focus on identifying and inhibiting such specific mechanisms to achieve the full potential of HSF1 inhibitors as next-generation anticancer therapeutics.

## 6. Future Prospects

The future of HSF1 inhibitors as next-generation chemotherapeutic drug primarily depends on their ability to specifically target HSF1. Solubility, toxicity, and bioavailability are other major factors that decide the fate of small molecule HSF1 inhibitors for therapeutic use. A prodrug strategy has resulted in the discovery of minnelide, a prodrug of triptolide as an impressive candidate with enhanced solubility and bioavailability. Furthermore, old molecules should be carefully re-examined and modified to transform them into appropriate clinical candidates.

There are only two HSF1 inhibitors (KRIBB11 and IHSF115) that can bind to HSF1 and affect subsequent transcription, rendering direct inhibition while most new inhibitors target HSF1 activation indirectly. Thus, the development of inhibitors that can directly target HSF1 can give better insights to the understanding of underlying mechanisms of HSF1 inhibition. As our understanding of biological pathways regulated by HSF1 in supporting cancers deepens, such as the role of the PIKK family, CDK9, and Pirin in the inhibition of the HSF1 stress pathway, identification of new chemotypes as potential candidate for cancer therapy will be easier. Notwithstanding the mechanism of action, many identified lead molecules can work in synergy with traditional therapies, which is promising because most current treatment strategies are not only based on single agent treatments but are also based on a combination of drugs.

## Figures and Tables

**Table 1:** Post translational modification of HSF1.

Repressive Modifications		Activating Modification	
Post Translational modification	Residue	Post Translational modification	Residue
Acetylation	K80	Phosphorylation	T142
Acetylation	K118	Phosphorylation	S195
Phosphorylation	S121	Acetylation	K208
Sumoylation	K293	Phosphorylation	S230
Phosphorylation	S303	Acetylation	K298
Phosphorylation	S307	Sumoylation	S320
Phosphorylation	S363	Phosphorylation	S326
		Sumoylation	S333
		Phosphorylation	S419

**Table 2:** List of HSF1 inhibitors of natural origin.

Compound Name and no.	Chemical Class	Structure	Mechanism of Action
Quercetin **1**	Flavonoid	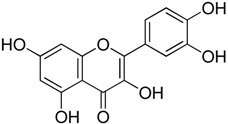	Inhibition of phosphorylation and downregulation of HSF1
Stresgenin B **2**	Carboxamide	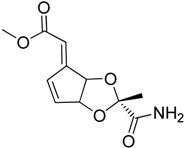	Not identified
Triplotide **3**	Diterpene Epoxide	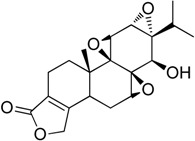	Inhibits transcriptional activity
Cantharidin **5**	Terpenoid	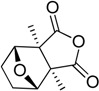	Inhibits HSF1 transcriptional activity
Fisetin **6**	Flavonoid	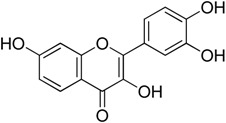	Blocks binding of HSF1 to the HSP70 promoter
Rocaglamide A **7**	Flavaglines	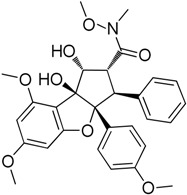	Diminishes promoter binding activity of HSF1
2,4-Bis(4-hydroxy benzyl)Phenol **8**	Phenol	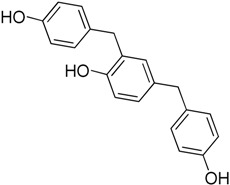	Dephosphorylation and degradation of HSF1
CL-43 **9**	Cardenolide	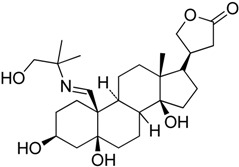	Not defined

**Table 3:** List of synthetic HSF1 inhibitors.

Compound Name and no.	Chemical Class	Structure	Mechanism of Action
KNK437 **13**	Lactam	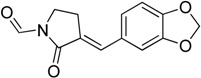	Blocks HSF1-mediated transcription and induces apoptosis
NZ-28 **14**	Emetine	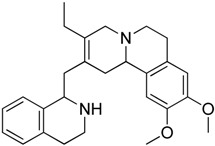	Inhibition of HSP mRNA translation
KRIBB11 **16**	Pyridinediamine	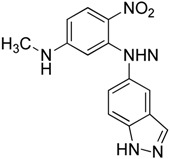	Binds to HSF1
PW3405 **17**	Anthraquinone	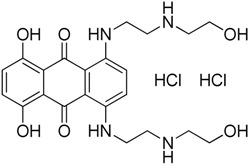	Inhibit phosphorylation of HSF1
I_HSF_115 **18**	Thiazole Acrylamide	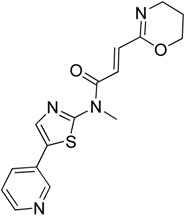	Binds to DBD of HSF1 and inhibits the transcriptional activity
4,6-Disubstituted Pyrimidine **19**	Pyrimidine	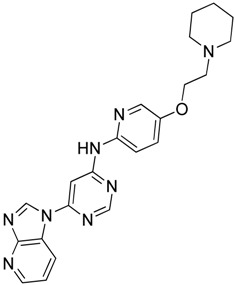	Post translational phosphorylation
CCT251236 **20**	Bisamide	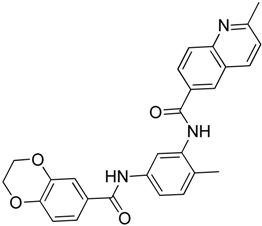	Under investigation
α−Acyl amino Carboxamides **21**	Aminocarboxamides	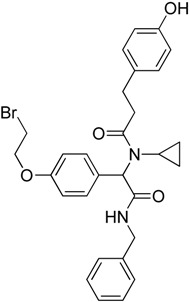	Destabilization of HSF1 protein and impaired translation of HSF1 mRNA
